# Community human settlement and self-rated health among older adults: the mediating role of sleep quality

**DOI:** 10.3389/fpubh.2026.1820032

**Published:** 2026-03-23

**Authors:** Chen Li, Chen Zhuo

**Affiliations:** 1 School of Management, Shanghai University of Engineering Science, Shanghai, China; 2 Faculty of Law and Justice, University of New South Wales, Sydney, NSW, Australia

**Keywords:** community human settlement quality, entropy method, older adults, self-rated health, Shanghai

## Abstract

**Introduction:**

Against the backdrop of rapid population aging, the community human settlement, as a crucial social environmental factor influencing the health levels of older adults, has increasingly become a focal point in public health research.

**Methods:**

Based on survey data from 2,113 older adults in Shanghai, China, this study employed the entropy method to construct a multi-dimensional community human settlement evaluation system encompassing the physical environment, social environment, and service accessibility. Multiple regression, instrumental variable methods, and mediation effect models were utilized to systematically analyze the pathways of influence and mechanisms of the community human settlement on the self-rated health of older adults.

**Results:**

The quality of the community human settlement was significantly positively correlated with the self-rated health levels of older adults, with community convenience and neighborhood support playing particularly prominent roles. Sleep quality exerted a significant mediating effect in this relationship, explaining 46% of the total effect. Among different groups, older adults with low income, those not married, and those with higher education levels were more sensitive to changes in the community human settlement, exhibiting stronger heterogeneity in health responses.

**Discussion:**

From the perspectives of ecological systems theory and the social determinants of health, this study reveals the psycho-physiological mechanism through which the community environment influences the health of older adults via sleep quality. It identifies environmentally sensitive high-risk groups, providing a theoretical basis and practical pathways for precise interventions in the construction of age-friendly communities.

## 1 Introduction

The world today is facing an unprecedented demographic transition, with population aging becoming a global challenge. The United Nations (2025) predicts that by the mid-21st century, over half of all deaths globally will occur among people aged 80 and over, compared to only 17% in 1995. As one of the developing countries with the fastest aging population, China is confronting the severe reality of “getting old before getting rich” (1). By the end of 2025, China’s population aged 60 and over reached 323.38 million, of which 223.65 million were aged 65 and over, an increase of 13.07 million and 3.42 million, respectively, compared to the previous year. The proportion of the population aged 60 and above accounted for 23.02% of the total population, and those aged 65 and above accounted for 15.92% (2). With the intensifying trends of advanced aging and empty-nest households, effectively improving the health level of older adults has become a core issue affecting sustainable social development and the stability of the national healthcare system.

In research on healthy aging, scientifically measuring the health status of older adults is a prerequisite for formulating effective intervention strategies. Among numerous assessment indicators, Self-Rated Health (SRH), due to its comprehensive reflection of an individual’s physiological function, psychological state, and social adaptability, is widely regarded as a valid indicator for predicting health outcomes and mortality risk in older adults (3, 4). SRH is not only a commonly used outcome variable in epidemiological studies but is also applied to monitor population health inequalities. Inyangetuk et al., using the Population Health Performance Index (PHPI), comprehensively assessed the average level of SRH and its inequality related to income and education across 21 regions in Sweden. They found a higher proportion of poor SRH in northern regions with significant regional differences, indicating that SRH as a monitoring tool can reveal the influence of structural social determinants (5).

In the study of SRH measurement, scholars have gradually realized that an individual’s evaluation of their own health is not a simple reflection of physiological status but a complex cognitive construction process. Early research primarily focused on constructing statistical explanatory models of SRH, exploring its association with sociodemographic factors and health indicators. Fylkenes and Forde, through an analysis of the Norwegian population, identified multidimensional factors influencing SRH, including physical health status, psychological state, and degree of social participation (6); Moum further expanded research in this area, systematically examining the distribution characteristics of SRH among Norwegian adults and its relationship with objective health indicators, providing an empirical basis for understanding sociodemographic differences in SRH (7). Building on this research, Knäuper and Turner proposed a cognitive process model of SRH, systematically describing the psychological process from question presentation to individual evaluation. The model begins with the premise that an individual’s judgment is based on an internal representation of their health, which integrates semantic knowledge, episodic memory, and perceptions of health trajectories. The model also incorporates the moderating effects of sociodemographic characteristics and personality factors on the evaluation process, emphasizing that different groups may form differentiated SRH results when facing the same health issues. Although the primary focus of that study was questionnaire design and it did not delve deeply into each stage of the evaluation process, it provided an important cognitive psychology perspective for understanding the formation mechanism of SRH (8).

Further, Marja Jylhä proposed a more systematic stage model of self-rated health, dividing the self-evaluation process into three progressive steps: First, the individual needs to understand the meaning of the concept of “health” and identify what should be considered components of “my health”; Second, the individual needs to comprehensively consider the relative importance of these components and their manifestation in the current state; Finally, the individual must select the most appropriate option from the pre-set scale levels to summarize the overall judgment (9). Therefore, the final SRH outcome depends not only on the individual’s understanding of “health” and their scrutiny of the components of “my health” but is also profoundly influenced by the socio-cultural context in which these health components are embedded and the way the pre-set scale is used. Jylhä’s model provides a more complete theoretical framework for understanding the formation mechanism of SRH and lays a theoretical foundation for subsequent explorations of how external environmental factors shape SRH outcomes by influencing an individual’s health cognition process.

Based on understanding the formation mechanism of SRH, scholars have further explored psychological and behavioral factors influencing SRH. Vučković et al. (10), drawing on Self-Determination Theory (SDT), found that exercise motivation mediates the relationship between perceived quality of sports facilities and SRH. Through structural equation modeling analysis of 546 adult exercisers, they validated the sequential mediation path of “service quality → exercise motivation → physical activity → SRH,” demonstrating that the external environment can indirectly improve health perceptions by stimulating intrinsic motivation, providing empirical support for the psychological mechanisms underlying the environment-health relationship.

Existing research indicates that factors influencing SRH among Chinese older adults are multidimensional, including demographic variables (e.g., age, gender, household registration), socioeconomic conditions, individual behaviors (e.g., social participation), and accessibility to medical resources, with significant health status differences existing among different groups (11, 12). Notably, the relationship between social participation and SRH is not unidirectional but involves dynamic interaction, with mutual influence and reinforcement (13).

In recent years, the community environment as a “social determinant of health” has gained increasing attention. The influence pathways of the community environment on health can be unfolded across three dimensions: first, behavior shaping, for example, walking facilities and green space area affecting physical activity levels; second, psychological regulation, such as the mitigating effect of neighborhood trust and safety on stress levels; and third, physiological exposure, such as the direct impact of environmental factors like air pollution and noise on physical health (14–16). Existing empirical studies show that good community greening levels are significantly positively correlated with SRH among older adults, with health effects differing across income and gender groups (17); the improvement of age-friendly facilities in communities, such as barrier-free design and optimized lighting, can effectively enhance older adults’ satisfaction with their living environment and subjective health levels (18). At the service resource level, the accessibility of community care services significantly improves the quality of life for urban older adults (19), and improvements in living conditions have also been found to alleviate depressive symptoms, particularly with more pronounced effects among psychologically vulnerable older groups (20). Furthermore, the community environment has been shown to moderate the impact of the household environment on mental health, further highlighting its mediating function in the health mechanisms of older adults (21).

It is noteworthy that the heterogeneity within the older population makes the aforementioned influence mechanisms more complex. Research focusing on older adults with disabilities found that the influence of socioeconomic status on SRH is not a simple direct effect but operates through a series of “reserve capacity” factors. Park and Ahn, analyzing data from the Korea Welfare Panel Study on disabled individuals aged 65 and over, found that interpersonal resources (social support satisfaction) and psychological resources (self-esteem) played significant mediating roles between socioeconomic status and SRH, while material resources (public service use) showed a negative association due to a “compensatory activation” mechanism (22). This finding not only confirms the critical role of psycho-social mechanisms in health pathways but also suggests that for older adults with different health conditions, the pattern of the relationship between environment and health may differ fundamentally, further highlighting the necessity of conducting group heterogeneity analysis.

Although the aforementioned studies reveal multiple pathways in the environment-health relationship, the direct applicability of their conclusions to Chinese older populations requires careful examination. Chinese society is characterized by a unique family-based cultural orientation, rapid urbanization, and the pension pressure of “getting old before getting rich.” The dependence on and perception of the community environment among Chinese older adults may exhibit different characteristics. For example, in the Chinese context, neighborhood mutual aid is not only a source of social support but also carries the emotional bonds of the traditional “acquaintance society”; the accessibility of community services pertains not only to convenience but is also closely related to institutional differences in the allocation of medical resources. Therefore, while drawing on international theories, this paper emphasizes integrating the construction of the community human settlement indicator system with the actual conditions of mega-city communities in China, highlighting the synergistic effects of the soft environment (neighborhood mutual aid, community participation) and the hard environment (housing quality, supporting facilities). This approach aims to more accurately reveal the social determinants of health among Chinese older adults and their pathways of action.

Based on the above analysis, this paper intends to make marginal contributions in the following three aspects: First, constructing an entropy method indicator system covering multiple dimensions such as the community physical environment, service accessibility, community facilities, and human settlement soft environment, serving the purpose of refined community governance in the context of mega-cities and providing methodological references for related research; Second, empirically testing, within the Chinese social context, the mediating role of sleep quality in the process where the community human settlement influences the SRH of older adults, thereby revealing the intrinsic pathway of the environment-health association from a psycho-physiological mechanism perspective; Third, based on the socioeconomic status, living conditions, and advanced age characteristics of the older group, conducting heterogeneity analysis to identify environmentally sensitive populations, providing a more precise basis for policy support and resource allocation for the construction of “age-friendly communities.”

## 2 Research hypotheses and theoretical framework

Systematically exploring the key factors influencing the self-rated health of older adults holds significant theoretical value and practical significance for optimizing healthy aging strategies and formulating differentiated intervention pathways. Among the multiple factors influencing health, the quality of the community environment has increasingly emerged as a core variable in recent years. Based on Ecological Systems Theory, individual health is a product of the interaction within multi-level ecosystems, constrained not only by micro-level physiological and psychological factors but also profoundly influenced by the meso-level social environment, particularly the community’s physical and social characteristics (23). For older adults with limited daily activity radii and strong resource dependence, the role of the community environment in maintaining their health is particularly crucial. A high-quality community environment—including clean living spaces, convenient mobility conditions, complete service facilities, safe security levels, and harmonious neighborly relations—not only helps promote regular exercise, medical service utilization, and social interaction, but also enhances life satisfaction and psychological well-being, thereby directly improving their health self-assessment. According to the Environment-Behavior-Health Model, the impact of the community environment on health often occurs indirectly through individual behavioral pathways (24). For example, communities with high green coverage, accessible public spaces, and good traffic safety often encourage older adults to participate more frequently in outdoor activities and social interactions, helping enhance physical fitness, delay the aging process, and alleviate negative psychological emotions such as loneliness and anxiety. Therefore, this paper proposes:

Research Hypothesis 1: The quality of the community human settlement has a significant positive impact on the self-rated health of older adults. That is, the higher the quality of the community human settlement, the higher the level of self-rated health among older adults.

Furthermore, the Social Determinants of Health (SDOH) theory emphasizes that sociodemographic characteristics, as structural variables, not only directly affect individual health but may also moderate the effect pathways of other health determinants (25). For the older population, their social structure characteristics (such as age, marital status, years of education, income level, etc.) play an important role in determining health behaviors, resource acquisition capabilities, and environmental adaptability, potentially leading to heterogeneity in the strength of the effect of community environment quality on health outcomes.

Specifically, older adults who are not married, lacking the emotional support and daily care of a spouse, face a higher risk of social isolation. The community environment plays a role as a substitute support system in their daily lives, making their health status more susceptible to the quality of the community environment; Older adults with higher education generally possess greater abilities in health awareness, information acquisition, and identifying and utilizing community resources, enabling them to better recognize and benefit from high-quality community environments; At the income level, low-income older adults, constrained by economic resources, have limited housing choices and rely more heavily on community support systems for their livelihood, thus showing stronger health responses to improvements in the quality of their living environment. Therefore, this paper further proposes:

Research Hypothesis 2: There is significant group heterogeneity in the effects of older adults’ age, marital status, education level, and income level on the impact of community human settlement quality on self-rated health.

Building on the above, further examining the mediating mechanism helps reveal the intrinsic pathway through which the community environment affects health. Sleep quality is an important physiological and psychological channel connecting the external environment and an individual’s health status. According to the Social Ecological Model (26), factors within the community environment such as noise, air quality, residential density, lighting conditions, and sense of safety can affect an individual’s mental stress and physiological rhythms, thereby influencing their sleep quality. For older adults, sleep is a critical process for maintaining bodily repair, immune function, and cognitive status. Good sleep quality not only improves physical condition but also enhances their sense of control over daily life and health perception. Conversely, a poor community environment can easily trigger sleep disorders, leading to negative health consequences such as fatigue, mood swings, and cognitive decline. Therefore, it is reasonable to infer that the effect of the community environment on the self-rated health of older adults may be partially realized through the mediating mechanism of sleep quality. Hence, we propose:

Research Hypothesis 3: Sleep quality plays a partial mediating role between community human settlement quality and the self-rated health of older adults. That is, the community human settlement indirectly improves the self-rated health level of older adults by enhancing their sleep quality.

**Figure 1:**
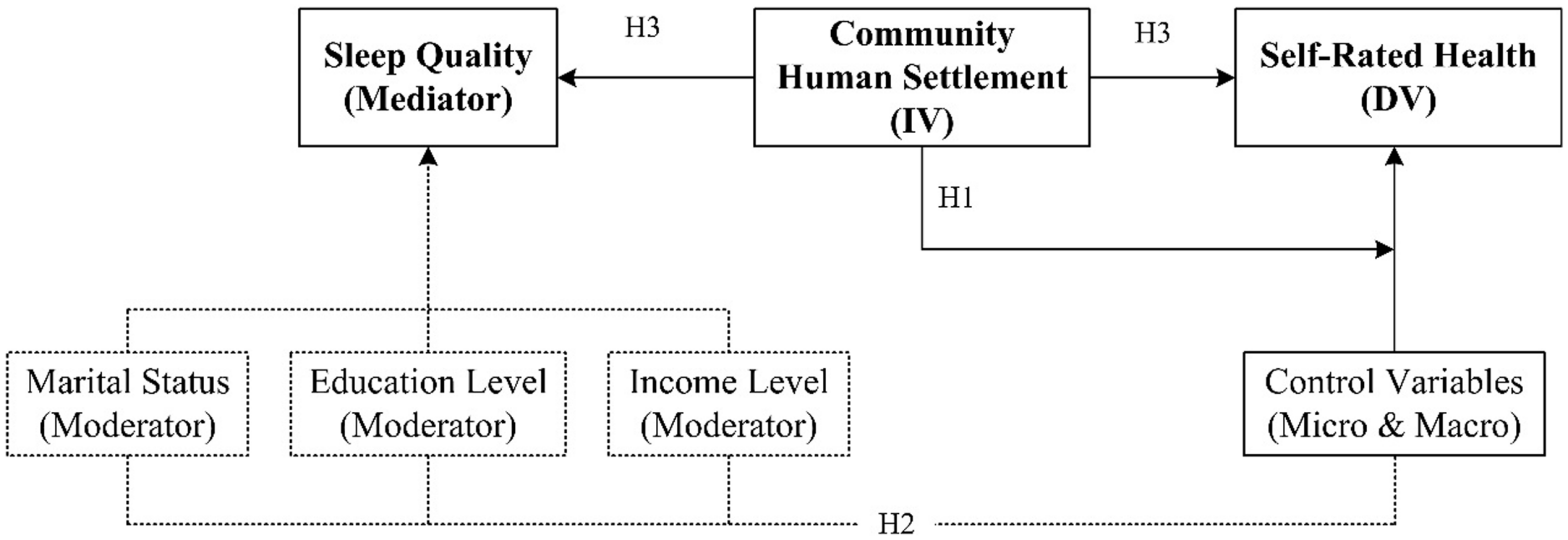
Theoretical framework of the study. Solid arrows represent causal paths (H1: Direct effect; H3: Mediating effect via sleep quality). Dashed arrows indicate moderating effects (H2) and statistical control (control variables). Moderators are hypothesized to influence the strength of the main path (community human settlement → self-rated health).

The three hypotheses above constitute the overall theoretical framework of this study: Hypothesis 1 tests the direct effect of the community human settlement on self-rated health; Hypothesis 2 tests the moderating effect of social structure variables (marriage, education level, income level) on this direct effect; Hypothesis 3 tests the mediating path of sleep quality between the two. The three hypotheses are interrelated, collectively revealing how and through what mechanisms the community environment affects the health of older adults, while also identifying which groups are more sensitive to this influence (Figure 1).

## 3 Data and methods

### 3.1 Overview of the study area

Shanghai is located at 31°14′North latitude and 121°29′East longitude, on the west coast of the Pacific Ocean and the eastern edge of the Asian continent. It is part of the alluvial plain of the Yangtze River Delta in China. Shanghai has an average temperature of 18.5 °C, 1489.7 h of sunshine, and 1414.2 mm of precipitation. The city’s administrative area is 6340.5 square kilometers, accounting for 0.06% of China’s total area (27). Shanghai governs 16 districts: Pudong New Area, Huangpu, Xuhui, Changning, Jing’an, Putuo, Hongkou, Yangpu, Minhang, Baoshan, Jiading, Jinshan, Songjiang, Qingpu, Fengxian, and Chongming. In 2023, Shanghai’s permanent resident population was 24.8745 million, of which the registered population was 15.1639 million, and the registered population aged 60 and over accounted for 37.37% (28).

### 3.2 Data sources and variable selection

#### 3.2.1 Data sources

The paper’s data originate from field surveys conducted by the author’s team in Shanghai. In June 2025, the research team conducted a pilot survey on older adults aged 60 and over in Shanghai’s Songjiang District, testing the reliability and validity of the pilot questionnaire. Based on questionnaire refinement, a formal survey of older adults in all 16 districts of Shanghai was conducted in July 2025. Using a random sampling method, 2,430 questionnaires were distributed. After data cleaning and excluding invalid questionnaires, 2,113 valid questionnaires were collected, yielding an effective rate of 86.95%. The construction of our multi-dimensional community human settlement evaluation system, encompassing physical environment, social environment, and service accessibility, aligns with recent methodological advances in measuring complex socio-economic phenomena at the micro level. For instance, studies examining the impact of artificial intelligence on corporate human capital structures have similarly employed composite indices based on multiple indicators to capture nuanced organizational changes (29). Likewise, research on industrial robots and firm innovation performance has demonstrated the importance of constructing comprehensive measurement frameworks that account for both quantity and quality dimensions of the outcome variable (30). Drawing on these methodological precedents, our entropy-based approach provides a robust foundation for analyzing the multi-faceted relationship between community environment and older health outcomes.

Micro-level data for the dependent variable, core explanatory variable, mediating variable, instrumental variable, and control variables were all obtained from the questionnaire survey. Macro-level control variables were calculated using data from the Shanghai Statistical Yearbook (28) and the Shanghai Older Care Service Platform (31). Considering that the older population data targets the permanent resident population, while the Shanghai Statistical Yearbook provides registered older population data, when calculating indicators such as the number of older care institutions per ten thousand older adults, the number of meal assistance service centers per ten thousand older adults, the number of day care centers per ten thousand older adults, and the number of activity rooms per ten thousand older adults, we used data on the permanent resident older population from the seventh national population census (32).

#### 3.2.2 Variables

##### 3.2.2.1 Dependent variable

The dependent variable is self-rated health of older adults, measured on a 1–5 scale. “Very poor,” “relatively poor,” “average,” “relatively good,” and “very good” self-rated health were assigned 1, 2, 3, 4, and 5 points, respectively. Higher scores indicate better self-rated health. In the robustness test section, self-rated health was converted into a dummy variable: “very poor,” “relatively poor,” and “average” were assigned 0 (indicating poor self-rated health), while “relatively good” and “very good” were assigned 1 (indicating good self-rated health). The robustness test also replaced self-rated health with pain level, where “no pain,” “occasional pain,” “frequent pain but not severe,” and “frequent pain and severe” were represented by 1, 2, 3, and 4 points, respectively. Higher scores indicate more severe pain.

##### 3.2.2.2 Core explanatory variable

Core Explanatory Variable: Community Human Settlement. The community human settlement is the specific embodiment of the human settlement system at the community level. Its theoretical roots can be traced back to the science of Ekistics, proposed by Greek architect C. A. Doxiadis. This theory analyzes human settlements into two major elements: “Content”——namely, humans and the society they form; and “Container”——namely, the tangible settlements composed of natural or man-made elements and their surrounding environment (33). Building on this foundation, Wu Liangyong, integrating China’s national conditions, established the Sciences of Human Settlements. He further divided human settlements into five systems (natural system, human system, social system, residential system, support system) and five levels (global, regional, urban, community, architectural), pointing out that the sustainable development of human settlements must follow five principles: ecological, economic, technological, social, and cultural (34).

**Figure 2:**
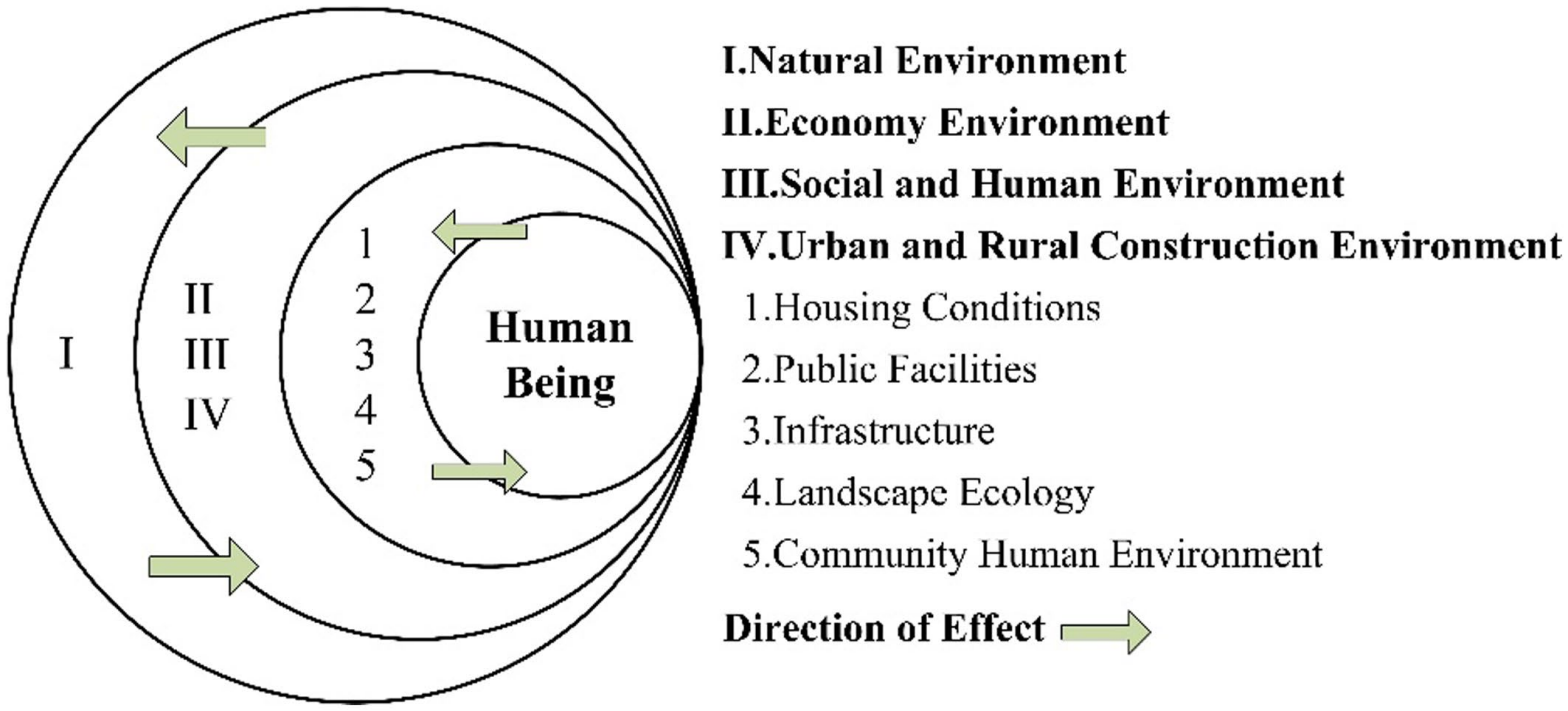
Structural diagram of the small-town human settlement system. Ning Yuemin et al. (36).

In Chinese academia, research on human settlements has undergone a deepening process from macro-level measurement to micro-level evaluation. Li Xueming et al. measured the quality of the urban human settlement environment from a macro perspective (35); Ning Yuemin et al. focused on the small town community level, constructing a human-centered structural model of the human settlement system (Figure 2). This model places humans at the center, with the immediate outer ring representing the human settlement environment in a narrow sense, together forming a broad human settlement system with the outer layers. This provides an important methodological foundation for evaluating the community human settlement environment (36).

Based on the aforementioned theoretical context and localized exploration, combined with the actual characteristics of mega-city communities in Shanghai, this paper constructs the community human settlement evaluation indicator system from the following four dimensions:

1. Housing Quality: Corresponds to the “container” element in Ekistics, focusing on the safety, comfort, and barrier-free accessibility of the living space. It reflects the basic residential security for residents.
2. Community Environment and Supporting Facilities: Also falls under the “container” category, encompassing internal community roads, lighting, leisure and fitness facilities, and ecological environment. It reflects the quality of the community’s physical space.
3. Community Facilities and Their Convenience: Corresponds to the “support system” in the Sciences of Human Settlements, reflecting residents’ accessibility to and efficiency of external resources such as medical care, daily life services, and public transportation.
4. Community Human Settlement Soft Environment: Corresponds to the “social system” and “human system,” emphasizing social capital elements such as neighborhood mutual aid and community participation. It reflects the community’s cohesion and social support function.

These four dimensions systematically cover the core elements emphasized by Ekistics and the Sciences of Human Settlements—physical environment, support systems, and social interaction—ensuring that the indicator selection has a theoretical foundation while also meeting the actual needs of the older group.

From an operationalization perspective, the four dimensions of the community human settlement are specifically measured as follows:

First, Housing Quality involves three questions: 1. Are you satisfied with the safety of your housing (fire prevention, theft prevention, safe evacuation, etc.)? 2. Are you satisfied with the comfort of your housing (temperature, humidity, ventilation, lighting)? 3. Are you satisfied with the barrier-free design of your residence (elevators, ramps, and other accessible facilities)?
Second, Community Environment and Supporting Facilities involves four questions: 1. Are you satisfied with the community road design? 2. Are you satisfied with the community lighting facilities? 3. Are you satisfied with the community leisure and fitness facilities (parks near the community, sports and fitness equipment)? 4. Are you satisfied with the environmental comfort of your community (ecological factors such as noise, air quality)?
Third, Community Facilities and Their Convenience involves three questions: 1. Are you satisfied with the accessibility and quality of community medical services? 2. Are you satisfied with the daily living facilities near the community (shops, supermarkets, markets, service centers for the older, etc.)? 3. Are you satisfied with the convenience of community public transportation facilities?
Fourth, Community Human Settlement Soft Environment involves two questions: 1. Are you satisfied with the mutual assistance situation among neighbors in your community? 2. Are you satisfied with the community activities (opportunities for older adults to participate in activities organized by the community)?

All 12 questions above use a Likert 5-level scale for satisfaction scoring (1 = Very Dissatisfied, 5 = Very Satisfied). Higher scores indicate higher satisfaction. The core explanatory variable “community human settlement” is derived by comprehensively weighting the 12 indicators and calculating a composite score using the entropy method, reflecting the older adults’ overall evaluation of their community human settlement.

##### 3.2.2.3 Mediating variable

The mediating variable is sleep quality, measured on a 1–5 scale. Higher scores indicate better sleep quality. Sleep is fundamental for individuals to maintain normal physiological functions and social activities. Poor sleep conditions, such as excessively short or long sleep duration, can affect multiple aspects of an individual’s life, thereby influencing self-rated health (37).

##### 3.2.2.4 Control variables

Control variables include both micro-level and macro-level aspects. Micro-level control variables consist of individual attributes (gender, age, marital status, ethnicity, years of education), personal habits (smoking status, drinking status, weekly exercise frequency), and economic factors (satisfaction with income sources, whether children provide financial support). The specific assignment methods are: For gender, male is assigned 1, female 0; For marital status, married is assigned 1, not married (divorced, widowed, unmarried, etc.) is assigned 0; For ethnicity, ethnic minorities are assigned 1, Han ethnicity is assigned 0; Years of education were assigned based on China’s educational stage durations: no schooling = 0, primary school = 6, junior high school = 9, senior high school = 12, junior college = 15, undergraduate = 16. For smoking status, smoker = 1, non-smoker = 0; For drinking status, drinker = 1, non-drinker = 0; For weekly exercise frequency, almost never = 0, 1–2 times per week = 1, 3–4 times per week = 2, 5 times or more per week = 3. For satisfaction with income sources, very dissatisfied = 1, dissatisfied = 2, moderately satisfied = 3, satisfied = 4, very satisfied = 5; for whether children provide financial support, providing support = 1, not providing support = 0.

Macro-level control variables consist of the number of health institutions per ten thousand people, the number of medical personnel per ten thousand people, the number of older care institutions per ten thousand older adults, the number of meal assistance service centers per ten thousand older adults, the number of day care centers per ten thousand older adults, and the number of activity rooms per ten thousand older adults. The number of health institutions per ten thousand people was calculated by dividing the number of health institutions in each Shanghai district by the permanent resident population; The number of medical personnel per ten thousand people was calculated by dividing the number of medical personnel (practicing (assistant) physicians plus registered nurses) in each district by the permanent resident population; The number of older care institutions per ten thousand older adults was calculated by aggregating data on older care institutions at the sub-district level provided by the Shanghai Older Care Service Platform and dividing by the permanent resident older population data from the seventh census; The number of meal assistance service centers per ten thousand older adults, the number of day care centers per ten thousand older adults, and the number of activity rooms per ten thousand older adults were similarly calculated by aggregating corresponding sub-district data from the Shanghai Older Care Service Platform and dividing by the permanent resident older population data. All macro-level control variables are continuous variables.

### 3.3 Research methods

#### 3.3.1 Entropy method

This study employs the entropy method to construct the core explanatory variable, community human settlement. This method plays a significant role in comprehensive evaluation and is a commonly used research method (38–41). As an objective weighting method, the entropy method effectively avoids the interference of subjective factors in weight determination. Its weights are calculated entirely based on the dispersion degree (entropy value) of the data, reflecting the variation information of each indicator among samples. In this study, all community human settlement indicators are positive indicators and have undergone regularization processing, eliminating dimensional influence and ensuring the objectivity and comparability of the weights. Concurrently, the large sample size (N = 2,113) covering all 16 districts of Shanghai ensures relatively stable data distribution, helping to enhance the reliability of weight estimation. Nevertheless, the entropy method still has certain limitations: it is sensitive to extreme values. Although this study mitigated the impact of extreme values through data cleaning and regularization, if systematic bias exists in the sample, it could still affect the stability of the weights. Future research could combine structural equation modeling or factor analysis to further validate the theoretical structure of each dimension of the community human settlement and their differentiated pathways affecting the health of older adults.

Calculation steps of the entropy method:

Firstly, the raw data is standardized. Since all the indicators related to community human settlement are positive indicators, all 12 indicators are subjected to normalization.


$$X{'}_{\mathrm{ij}}=\frac{X_{\mathrm{ij}}-\min(X_{\mathrm{ij}})}{\max(X_{\mathrm{ij}})-\min(X_{\mathrm{ij}})}$$

Among them, *X_ij_’* refers to the assigned value of each individual’s 12 indicators related to community human settlement in the survey questionnaire, where *i* = 1, 2, …, 2,113; *j* = 1, 2, …, 12.

Next, calculate the weights.

(1) Calculate the ratio of the evaluation indicator for the *i*-*^th^* survey object under the *j-^th^* indicator, *P_ij_*:

$$Pi_{j}=\frac{X^{\prime }i_{j}}{\sum_{i=1}^{2113}X{'}_{\mathrm{ij}}}$$

(2) Calculate the entropy value of the *j-^th^* evaluation indicator, *E_j_*:

$$E_{j}=-\frac{1}{\ln2113}\sum_{i=1}^{2113}P_{\mathrm{ij}}\ln(P_{\mathrm{ij}})$$

(3) Calculate the weight of the *j-^th^* evaluation indicator, *w_j_*:

$$w_{j}=\frac{1-E_{j}}{\sum_{j=1}^{12}(1-E_{j})}$$

Finally, calculate the composite score of the *i-^th^* survey object for community human settlement.


$$\text{composite}=\sum_{j=1}^{12}w_{j}X_{\mathrm{ij}}^{\prime }$$

#### 3.3.2 Regression model

Since the dependent variable, self-rated health of the older, is a continuous variable, a multiple linear regression model is constructed, and the specific formula is as follows:


$$Y=\beta_{0}+\beta_{1}X_{1}+\sum \beta_{k}X_{k}+\varepsilon_{i}$$

In this model, *Y* represents the self-rated health of the older, *X_1_* is the comprehensive score of community human settlement, *X_k_* represents the control variables, *β_0_* is the intercept, *β_1_* is the regression coefficient of community human settlement, *β_k_* represents the regression coefficients of the control variables, and *ε_i_* is the residual term.

Considering the potential endogeneity issue of the independent variables, in order to obtain consistent estimates and accurately reflect the causal relationship between community human settlement and the self-rated health of the older, the study uses the two-stage least squares method. The specific procedure is as follows:

In the first stage of the regression, the endogenous variable, community human settlement, is regressed on the instrumental variable, the evaluation of the community aging-friendly renovation policy, to obtain the fitted values, that is:


$$X_{1}=\alpha_{0}+\alpha_{1}Z_{1}+\sum \alpha_{k}X_{k}+u_{i}$$

$${X_{1}}^{'}=\alpha_{0}+\alpha_{1}Z_{1}+\sum \alpha_{k}X_{k}$$

Where, *X_1_’* is the fitted value of the community human settlement score, *Z_1_* is the instrumental variable, *α_0_* is the intercept, *α_1_* is the regression coefficient of the instrumental variable, *α_k_* is the regression coefficient of the control variables, and *u_i_* is the error term.

In the second stage regression, replace the original variable with the fitted value and perform the second stage regression:


$$Y=\delta_{0}+\delta_{1}{X_{1}}^{'}+\sum \delta_{k}X_{k}+v_{i}$$

Where, *δ_0_* is the intercept, *δ_1_* is the regression coefficient of the fitted value of the community living environment, *δ_k_* is the regression coefficient of the control variables, and *v_i_* is the residual term.

#### 3.3.3 Mediation effect model

Using the three-step method to analyze the mediating effect of sleep quality in the relationship between community human settlement and older people self-reported health.

Step 1: Estimate the effect of the core explanatory variable, community human settlement(*X_1_*), on the dependent variable, older people self-reported health (*Y*).


$$Y=\beta_{10}+\beta_{11}X_{1}+\varepsilon_{1}$$

Step 2: Estimate the effect of the core explanatory variable, community human settlement(*X_1_*), on the mediator variable, sleep quality (*M*).


$$M=\beta_{20}+\beta_{21}X_{1}+\varepsilon_{2}$$

Step 3: Simultaneously include the core explanatory variable, community human settlement(*X_1_*), and the mediator variable, sleep quality (*M*), into the model.


$$Y=\beta_{30}+\beta_{31}X_{1}+\beta_{32}M+\varepsilon_{3}$$

In the above model, *β_11_* represents the total effect of the core explanatory variable *X_1_* (community human settlement) on the dependent variable Y. After adding the mediator variable *M* (sleep quality), the effect of *X_1_* on *Y*, denoted as *β_21_*, represents the direct effect. *β_21_* × *β_32_* represents the indirect effect. Based on the strength of the mediation effect, it can be classified into full mediation and partial mediation. Full mediation occurs when the regression coefficient of the core explanatory variable (community human settlement) on the dependent variable (self-rated health of the older) becomes insignificant after adding the mediator variable (sleep quality). Partial mediation occurs when, after adding the mediator variable, the regression coefficient of the core explanatory variable decreases compared to the model without the mediator variable, but remains statistically significant, indicating that the mediator variable only explains part of the effect of the independent variable.

## 4 Results and analysis

### 4.1 Baseline regression

The regression results show that, regardless of whether control variables are included, the community human settlement has a significant positive impact on the self-rated health of older adults, consistent with Research Hypothesis 1 (Table 1). Specifically, without controlling for any variables, a one-unit increase in the community human settlement composite score significantly increases the self-rated health score of older adults by 2.783 points, indicating a substantial independent contribution of community environment quality to health. After introducing micro-level control variables (Model 2), the coefficient decreases slightly to 2.215 but remains significant and influential, suggesting that individual attributes and lifestyle habits partially explain the relationship. When controlling only for macro-level variables (Model 3), the coefficient is 2.810, close to Model 1, indicating that the macro environment does not significantly weaken the effect of the community environment on SRH. Further introducing both macro and micro control variables in Model 4, the coefficient remains significant (2.233), demonstrating that the positive effect of community environment quality on the health of older adults is robust. This result aligns with the expectations of Ecological Systems Theory and the Environment-Behavior-Health Model, confirming that the community, as the meso-environment for older adults’ daily lives, plays an irreplaceable key role in health maintenance.

**Table 1:** Baseline regression results.

Variable	Model 1 SRH	Model 2 SRH	Model 3 SRH	Model 4 SRH
CHS	2.783***	2.215***	2.810***	2.233***
	(0.108)	(0.132)	(0.108)	(0.132)
Gender		0.113***		0.096**
		(0.042)		(0.042)
Age		−0.015***		−0.015***
		(0.003)		(0.003)
Marital status		0.151***		0.144***
		(0.044)		(0.044)
Race		0.127		0.129
		(0.088)		(0.088)
Years of education		0.001		0.004
		(0.004)		(0.004)
Smoking status		−0.011		−0.003
		(0.044)		(0.044)
Drinking status		−0.002		0.002
		(0.043)		(0.043)
Weekly exercise frequency		0.111***		0.111***
		(0.019)		(0.019)
Children provide financial support		−0.071*		−0.086**
		(0.037)		(0.038)
Satisfaction with income sources		0.178***		0.176***
		(0.025)		(0.025)
Health inst. per 10 k people			−0.083***	−0.077***
			(0.026)	(0.026)
Medical personnel per 10 k people			0.002***	0.001***
			(0.000)	(0.000)
Older people care inst. per 10 k older adults			0.209**	0.154*
			(0.090)	(0.087)
Meal assistance centers per 10 k older adults			−0.012	−0.015**
			(0.008)	(0.007)
Day care centers per 10 k older adults			0.024*	0.039***
			(0.014)	(0.014)
Activity Rooms per 10 k older adults			0.019**	0.017**
			(0.008)	(0.008)
Constant	1.682***	2.224***	1.121***	1.779***
	(0.075)	(0.223)	(0.202)	(0.292)
*N*	2,113	2,113	2,113	2,113
R^2^	0.239	0.295	0.246	0.302

***p < 0.01; **p < 0.05; *p < 0.1. Standard errors are in parentheses. CHS refers to community human settlement.

From the regression results of the control variables, multiple factors at both micro and macro levels significantly influence the self-rated health of older adults. Specifically, individuals who are male, married, younger, exercise more frequently, and have higher satisfaction with their income sources report significantly higher levels of self-rated health. Among these, the effects of exercise frequency and economic satisfaction are most pronounced, highlighting the crucial roles of health behavior and economic perception in health evaluation. Conversely, children providing financial support is negatively correlated with self-rated health, which may reflect a higher dependence on family resources among individuals with poorer health status. At the macro level, the number of medical personnel per ten thousand people, the number of older people care institutions, day care centers, and community activity rooms all positively impact the health of older adults, underscoring the functional value of public service provision in promoting health. However, the number of health institutions per ten thousand people and meal assistance service centers are negatively correlated with SRH, potentially revealing a passive configuration characteristic of health service supply, where increased service provision might be a response to poorer health status. Overall, comparisons between models indicate that both micro-level individual differences and macro-level resource environments significantly explain the self-rated health of older adults. Moreover, after incorporating multiple control factors, the significance and coefficient of the community human settlement remain stable, further highlighting its undeniable core position in the process of healthy aging.

Decomposing the core explanatory variable community human settlement into four secondary indicators—Housing Quality, Community Environment and Supporting Facilities, Community Facilities and Their Convenience, and Community Human Settlement Soft Environment—the regression analysis results show that all four secondary indicators have a significant positive impact on the self-rated health levels of older adults, with varying degrees of influence. After controlling for a series of individual characteristic variables and regional fixed effects, the regression coefficients for all four indicators passed the 1% significance test, indicating that improvements in each dimension of the human settlement environment contribute to enhancing the health perception of older adults (Table 2).

**Table 2:** Regression results by dimension.

Variables	Model 5	Model 6	Model 7	Model 8
	SRH	SRH	SRH	SRH
Housing quality	5.620***			
	(1.201)			
Community environment and facilities		4.938*** (0.729)		
Community facilities and convenience			6.368*** (0.926)	
Community human settlement soft environment				6.917*** (1.628)
Control variables	Yes	Yes	Yes	Yes
Regional fixed effects	Yes	Yes	Yes	Yes
Constant	1.870***	2.026***	1.932***	2.158***
	(0.337)	(0.273)	(0.290)	(0.273)
*N*	2,113	2,113	2,113	2,113
*_R_2*	0.276	0.275	0.267	0.254

***p < 0.01. Standard errors in parentheses.

Specifically, the Community Human Settlement Soft Environment has the most significant effect (coefficient 6.917, *p* < 0.01), suggesting that good neighborly mutual aid relationships and opportunities for community activity participation can significantly enhance older adults’ sense of social support and mental health, thereby strengthening their positive evaluation of health status. Next, the regression coefficient for Community Facilities and Their Convenience is 6.368 (*p* < 0.01), highlighting the critical role of medical service accessibility, completeness of living facilities, and public transportation convenience in improving the health perception of older adults. The regression coefficient for Housing Quality is 5.620 (*p* < 0.01), reflecting the importance of residential safety, comfort, and the completeness of barrier-free facilities for the physical health and daily living convenience of older adults. Although the Community Environment and Supporting Facilities has a relatively smaller impact (coefficient 4.938, *p* < 0.01), its role in improving the quality of the living ecological environment and leisure and fitness conditions, thereby enhancing the health perception of older adults, remains undeniable.

Overall, the multi-dimensional optimization of the community human settlement environment has a significant impact on the self-rated health of older adults. Particular attention should be paid to the systematic construction of the community soft environment and supporting service systems to more effectively promote the physical and mental health and well-being of the older population. This finding has important practical implications for advancing the construction of age-friendly communities and the formulation of healthy aging policies.

### 4.2 Endogeneity analysis

In Two-Stage Least Squares (2SLS) estimation, the instrumental variable must satisfy both the relevance and exogeneity conditions. Relevance requires the instrumental variable to be significantly correlated with the endogenous explanatory variable. Exogeneity requires the instrumental variable to influence the dependent variable only through the explanatory variable and be uncorrelated with the regression model’s error term. This study selects “Evaluation of community aging-friendly renovation policies” as the instrumental variable for the following reasons: First, the implementation quality and effectiveness of community aging-friendly renovation policies typically directly affect the degree of community environment improvement. Therefore, higher policy evaluation levels are more likely associated with better overall quality of the community human settlement, satisfying the condition of significant correlation between the instrument and the explanatory variable. Second, the implementation of community renovation policies is primarily driven by government departments and does not have a direct causal path with the individual health status of older adults. Simultaneously, as a subjective perception of older adults regarding the effects of community renovations, policy evaluation might be partially influenced by individual psychological states or health status. To mitigate the endogeneity risk introduced by individual subjective factors, the study controlled for variables related to older adults’ psychological attitudes and social support in the regression model, aiming to ensure that variations in the instrumental variable primarily reflect objective differences in policy implementation, thus better satisfying the exogeneity assumption.

Table 3, column (2), reports the first-stage regression results. The estimated coefficient for the instrumental variable “Evaluation of community aging-friendly renovation policies” is significantly positive, indicating a significant positive correlation with the quality of the community human settlement, meeting the relevance requirement for an instrumental variable. Further, replacing the original community human settlement variable with its fitted values obtained from the first-stage regression, the second-stage regression results in Table 3, column (3), show that the community human settlement has a significant positive impact on the self-rated health of older adults, supporting the conclusion that community environment improvement helps enhance the self-rated health of older adults. Regarding the validity test of the instrumental variable, the weak identification test result (Cragg-Donald Wald F statistic = 234.342) is significantly larger than the 10% maximal IV size, ruling out the weak instrument problem. As the model is exactly identified, overidentification tests cannot directly verify instrument exogeneity. To further indirectly verify exogeneity, Table 3, column (4), reports the results of a reduced-form regression. The results show that when the instrumental variable is directly included in the self-rated health equation, its coefficient is non-significant, consistent with the semi-reduced form test logic proposed by Sun and Chen, i.e., if the instrumental variable is uncorrelated with the error term, its coefficient in the original equation should be non-significant (42). Based on these tests, the use of the instrumental variable method effectively mitigates endogeneity concerns, and the baseline regression conclusions remain robustly valid. While these tests suggest that endogeneity concerns are substantially mitigated, the possibility of remaining bias cannot be entirely ruled given the cross-sectional nature of the data.

**Table 3:** Instrumental variable test.

Variables	First-stage regression	Second-stage regression	Semi-reduced form regression
	CHS	SRH	SRH
Instrumental variable	0.075***		0.016
	(0.005)		(0.280)
CHS		2.404***	2.188***
		(0.636)	(0.265)
Control variables	Yes	Yes	Yes
Regional fixed effects	Yes	Yes	Yes
Cragg-Donald Wald F statistic		234.342	
*N*	2,113	2,113	2,113
*_R_2*	0.511	0.307	0.307

***p < 0.01. Standard errors in parentheses.

### 4.3 Robustness tests

To verify the robustness of the baseline regression results, this paper reprocesses and substitutes the dependent variable from multiple dimensions, specifically including constructing a binary classification model, introducing an alternative health indicator, and adjusting the sample range, to further test the stability of the impact of the community human settlement on the health of older adults. The four sets of robustness regression results presented in Table 4 all show that improvements in the quality of the community human settlement consistently have a significant positive impact on the health of older adults, highly consistent with the baseline regression results.

**Table 4:** Robustness test results.

Variable	Model 9 (Probit) SRH_dummy	Model 10 Pain Level	Model 11 SRH (Excl. 1% tails)	Model 12 SRH (Excl. 5% tails)
Composite	3.132***	−0.997**	2.211***	1.595***
	(0.648)	(0.406)	(0.326)	(0.304)
Control variables	Yes	Yes	Yes	Yes
Regional fixed effects	Yes	Yes	Yes	Yes
Constant	−0.493	2.503***	1.776***	2.252***
	(0.572)	(0.391)	(0.277)	(0.358)
*N*	2,113	2,113	2071	1901
(Pseudo) R^2^		0.157	0.278	0.158

***p < 0.01; **p < 0.05. Standard errors in parentheses. Model 9 reports Probit coefficients, not marginal effects.

First, the self-rated health variable was recoded as a dummy variable (“Poor” = 0, “Good” = 1) and estimated using a Probit model. The results show that the marginal effect of the community human settlement is significantly positive (coefficient 3.132, significance level 1%), indicating that community environment improvement can significantly increase the probability of older adults being in a good health state. This result further strengthens the OLS baseline regression conclusion from a non-linear probability model perspective. Second, “Pain Level” was used as a substitute dependent variable for robustness testing. The results show that the regression coefficient for the community environment variable is −0.997, negative and significant at the 5% level, meaning that community environment improvement helps alleviate physiological discomfort among older adults, confirming its positive impact from another health dimension.

Third, to control for potential extreme value interference, this study re-ran the regression after excluding samples with health scores in the top 1% and bottom 1%. The results show that the coefficient for the community human settlement remains significantly positive (2.211, *p* < 0.01), which is essentially consistent with the result from Model 2 in the baseline regression (2.215), indicating that extreme values have a minor impact on the estimation results. After further expanding the exclusion range (removing the top and bottom 5% of samples), the coefficient for the community environment’s impact decreases slightly (1.595) but remains statistically significant at the 1% level, demonstrating that the research conclusion remains robust even under substantial sample adjustments.

The results of the above multi-dimensional robustness tests all support the core findings of the baseline regression, indicating that the positive impact of the community human settlement on the health of older adults is robust across different model specifications, variable substitutions, and sample processing methods. It also exhibits good statistical significance and explanatory power, further validating the reliability and generalizability of the research conclusions.

### 4.4 Heterogeneity analysis

Building on the confirmed robustness of the core findings, this study proceeds to examine potential heterogeneity by investigating whether the health effects of community human settlement vary across different subgroups of older adults. In the heterogeneity analysis of the impact of the community human settlement on the self-rated health of older adults, the results show that this impact is significantly positive across groups with different social structure characteristics, but the strength of the effect varies significantly, indicating heterogeneity in the health-promoting effect of community environment quality among groups (Table 5).

**Table 5:** Heterogeneity estimation results.

Model 13		Model 14		Model 15		
Variables	Married	Not married	Received higher education	Did not receive higher education	High- income group	Low- income group
CHS	2.084***	2.625***	2.757***	2.166***	1.517***	2.715***
	(0.318)	(0.437)	(0.587)	(0.330)	(0.369)	(0.340)
Control variables	Yes	Yes	Yes	Yes	Yes	Yes
Regional fixed effects	Yes	Yes	Yes	Yes	Yes	Yes
Constant	1.815***	1.405**	1.941	1.548***	2.390***	1.274**
	(0.385)	(0.517)	(1.940)	(0.262)	(0.747)	(0.468)
*N*	1,567	546	346	1767	1,013	1,100
*_R_2*	0.304	0.306	0.281	0.313	0.208	0.379

***p < 0.01; **p < 0.05. Standard errors in parentheses.

Specifically, among married older adults, the coefficient for the impact of the community human settlement on SRH is 2.084 (*p* < 0.001), while among those not married, it is 2.625 (*p* < 0.001), the latter being significantly higher. This confirms that older adults not married, lacking spousal support, rely more on the community to provide emotional connections and alternative mechanisms for daily care, making the community environment play a more critical role in their health maintenance. Regarding education level heterogeneity, the coefficient for those with higher education is 2.757 (*p* < 0.001), significantly higher than the 2.166 (*p* < 0.001) for those without higher education. This suggests that older adults with higher education levels may benefit more fully from high-quality community environments due to their stronger information acquisition capabilities, health awareness, and ability to utilize community resources. In income grouping, the estimated coefficient for the low-income group is 2.715 (*p* < 0.001), significantly higher than the 1.517 (*p* < 0.001) for the high-income group, further indicating that resource-constrained individuals have higher health sensitivity and dependence on community public services and environmental improvements. These findings align with the Social Determinants of Health theory, verifying that sociodemographic characteristics, as structural variables, not only directly affect the health status of older adults but also moderate the community environment-health pathway, providing empirical support for Research Hypothesis 2.

## 5 Further analyses: mediation mechanism test

In the mechanism analysis section, this paper further tests how the community human settlement influences the self-rated health level of older adults through the mediating variable of sleep quality. The empirical results are detailed in Table 6. The regression results show that the total effect of the community human settlement on the self-rated health of older adults is significant at 2.233 (*p* < 0.001), indicating that a good community environment overall helps improve the health perception of older adults. Further analyzing the mediation path reveals that the community human settlement has a significant positive impact on sleep quality (coefficient 2.494, *p* < 0.001), and the regression coefficient of sleep quality on self-rated health is also positive and significant (0.416, *p* < 0.001). After controlling for sleep quality, the direct effect of the community environment on self-rated health decreases to 1.196 (*p* < 0.001), remaining significant, which verifies the existence of a partial mediation effect. Calculating using the product of coefficients method, the indirect effect = 2.494 × 0.416 ≈ 1.038, accounting for approximately 46.49% (1.038/2.233) of the total effect. This indicates that sleep quality explains nearly half of the effect in the pathway through which the community human settlement influences the self-rated health of older adults.

**Table 6:** Mediation mechanism test results.

Variables	SRH	Sleep quality	SRH
CHS	2.233***	2.494***	1.196***
	(0.323)	(0.301)	(0.304)
Sleep quality			0.416***
			(0.044)
Control variables	Yes	Yes	Yes
Regional fixed effects	Yes	Yes	Yes
Constant	1.779***	1.738***	1.057***
	(0.267)	(0.351)	(0.243)
*N*	2,113	2,113	2,113
*_R_2*	0.307	0.282	0.434

***p < 0.01. Standard errors in parentheses.

The above results provide strong empirical support for Research Hypothesis 3, namely that sleep quality plays a partial mediating role between the community environment and the self-rated health of older adults. This finding aligns with the theoretical presuppositions of the Social Ecological Model and Stress-Recovery Theory, revealing a key physiological and psychological mechanism pathway through which the community environment affects health. Specifically, a good community environment can alleviate the mental stress of older adults and improve their sleep–wake rhythms, thereby enhancing their sleep quality and further promoting their health perception level. This mechanism analysis not only enriches the research connotation of the relationship between community environment and health but also provides directional insights for policy formulation——by optimizing sleep-related environmental factors such as community noise management, air quality, lighting conditions, and public security, it is possible to indirectly improve the health level of older adults, achieving more targeted health promotion interventions.

To ensure the robustness of the mediation effect estimation, this study further employed the bias-corrected Bootstrap method (with 5,000 resamples) to estimate the confidence intervals of the indirect effect. The results (Table 7) show that the mediation effect value of sleep quality is 1.038, with a Bootstrap standard error of 0.102, and its bias-corrected 95% confidence interval is [0.839, 1.237], which does not contain zero. This indicates that sleep quality plays a significant partial mediating role between the community human settlement and self-rated health. Furthermore, the Bootstrap confidence intervals for both the direct effect and the total effect also do not contain zero, further supporting the reliability of the partial mediation model.

**Table 7:** Bootstrap test results for the mediation effect.

Effect Type	Estimate	Bootstrap standard error	Bias-corrected 95% CI
Mediation effect (a × b)	1.038	0.102	[0.839, 1.237]
Direct effect (c’)	1.196	0.304	[0.600, 1.792]
Total effect (c)	2.233	0.323	[1.600, 2.866]

The number of Bootstrap resamples is 5,000; confidence intervals were calculated using the bias-corrected percentile method.

## 6 Discussion

This study engages in theoretical dialogue with Jylhä’s cognitive model of self-rated health. Jylhä pointed out that self-rated health originates from an individual’s active integration of internal bodily sensations and socio-cultural frameworks. This study incorporates the community human settlement as an external social environmental factor into that framework, confirming that community quality shapes the health perceptions of older adults by influencing the “interoceptive information” channel of sleep, thereby extending the application boundary of that theory in environmental health research.

Unlike Inyangetuk et al. who focus on macro-regional health inequalities, this study delves into the micro-level of the community, finding that groups such as low-income and not-married individuals are more sensitive to the community environment, deepening the understanding of structural health inequalities from an environmental sensitivity perspective. Compared with the behavioral path of “service quality → exercise motivation → physical activity” revealed by Vučković et al., this study reveals a psycho-physiological path of “community human settlement → sleep quality → self-rated health.” The two mechanisms operate in parallel and are complementary, with the sleep mediation showing stronger explanatory power among the older group (accounting for 46% of the total effect), reflecting the distinctiveness of this study. The mediating role of sleep quality identified in our research resonates with findings from urban economics, where directed technological change has been shown to mediate the relationship between urban density and carbon intensity (43). In both contexts, a key intervening variable—whether sleep quality or capital-saving technological change—serves as the critical pathway through which environmental or structural factors translate into individual or societal outcomes. This parallelism underscores the broader applicability of mediation frameworks in understanding how macro-level conditions shape micro-level results across different domains of social science research.

However, our study has the following limitations: First, the cross-sectional data cannot fully confirm causality; future research needs to adopt longitudinal designs. Second, the core variables are based on self-reports, which may involve measurement bias; future studies should incorporate objective environmental measurements and physiological indicators. Third, conclusions from a single city need validation in more regions. Fourth, only the mediation of sleep was examined; subsequent research should integrate multiple pathways such as psychological stress and social participation. Fifth, the PHPI method used by Inyangetuk et al. could be referenced to construct a community health performance index, providing more systematic decision-making tools for precise interventions.

## 7 Conclusion and policy recommendations

Based on survey data from 2,113 older adults in Shanghai, this study used the entropy method to construct a multi-dimensional community human settlement evaluation system and systematically tested the influence mechanism of the community environment on the self-rated health of older adults, yielding the following main conclusions:

First, the quality of the community human settlement has a significant positive impact on the self-rated health of older adults, and this effect remains robust in multi-dimensional models. Among the dimensions, the Community Human Settlement Soft Environment (neighborhood mutual aid, activity participation) and Facility Convenience (medical care, daily services, transportation) play the most prominent roles, indicating that social support and resource accessibility are core elements for enhancing the subjective health perception of older adults. This finding confirms, from the perspective of Ecological Systems Theory, the nested influence of the meso-environment on individual health: the community, as the primary setting for the daily activities of older adults, shapes health outcomes directly or indirectly through multiple pathways including behavior shaping, psychological regulation, and physiological exposure.

Second, there is significant group heterogeneity in the impact of the community environment on health. Older adults who are not married, have low income, or have received higher education are more sensitive to changes in the community environment, exhibiting stronger health responses. This result deepens the connotation of the Social Determinants of Health theory: social structure variables not only directly affect health levels but also alter the strength of environmental effects by moderating individuals’ dependence on and ability to utilize environmental resources. Those not married, lacking intra-family emotional and instrumental support, rely more on the community to provide alternative social networks; low-income groups, constrained by economic resources, have a higher dependence on community public services; while highly educated older adults, possessing stronger information acquisition and resource integration capabilities, are better at benefiting from high-quality environments. The above heterogeneity suggests that community health interventions need to shift from a “one-size-fits-all” approach to “precision targeting,” allocating resources according to the needs of different groups to achieve fairness and efficiency.

Third, sleep quality plays an important mediating role in the pathway through which the community human settlement influences self-rated health, explaining 46% of the total effect. This finding reveals a key psycho-physiological channel in the environment-health association: a good community human settlement (e.g., low noise, adequate lighting, safe order, pleasant landscape) can alleviate the mental stress of older adults, improve their sleep–wake rhythms, and thereby promote health perception by enhancing sleep quality. This mechanism not only provides empirical support for the Social Ecological Model but also suggests that sleep, as a mediating variable connecting the external environment and internal health, should be an important target for community health interventions.

Based on the above findings, this paper proposes the following policy recommendations and practical pathways:

1. Strengthen the Construction of Community Soft Environment and Supporting Services to Create an All-Age Friendly Living Circle. Efforts should focus on enhancing neighborhood mutual aid networks, opportunities for community activity participation, and the accessibility of medical and daily life services, shifting community building from “hardware improvement” to “emphasizing both hardware and software.” For example, by establishing community neighborhood centers, organizing regular cultural and sports activities for older adults, and optimizing the layout of community health service stations, the sense of social support and living convenience for older adults can be enhanced, thereby improving their overall health perception.
2. Implement Differentiated Precision Interventions, Prioritizing Coverage of Environmentally Sensitive High-Risk Groups. For older adults who are not married or have low income, investment in community care, visitation services, and age-friendly renovations should be increased, establishing a “community-family” complementary support system. For the highly educated older group, information platforms and health lectures can be used to guide them in fully utilizing community resources, leveraging their resource integration advantages. By identifying the needs characteristics of different groups, the benefits of environmental improvements can be maximized.
3. Integrate Sleep Health into Community Environmental Governance Goals, Establishing a Collaborative Promotion Mechanism of “Environment-Sleep-Health.” In community planning and renovation, environmental factors related to sleep, such as noise control, nighttime lighting, air quality, and public security conditions, should be systematically assessed. Quantitative standards should be formulated and incorporated into the evaluation systems for renovating old residential communities and constructing new ones. Simultaneously, sleep health education can be conducted at the community level to help older adults establish good sleep habits, forming a dual guarantee of environmental optimization and behavioral intervention.
4. Promote the Deep Integration of Community Environmental Governance with the Healthy Aging Strategy. It is recommended to incorporate the community human settlement index into the monitoring indicators for local aging cause development, establish regular assessment and dynamic feedback mechanisms, and promote the implementation of “Health in All Policies” at the grassroots level. Through multi-sectoral coordination (housing and urban–rural development, civil affairs, health, planning, etc.), health impact assessments should be embedded in the approval process for community construction projects to ensure that environmental improvement measures truly translate into health benefits for older adults.

In summary, from the perspectives of Ecological Systems Theory and the Social Determinants of Health, this study reveals the psycho-physiological mechanism through which the community environment influences the health of older adults via sleep quality. It identifies environmentally sensitive high-risk groups, providing a theoretical basis and precise intervention pathways for the construction of age-friendly communities. Future research could further integrate longitudinal data to explore the long-term health effects of changes in the community environment and incorporate more physiological indicators and objective environmental measurements to deepen the understanding of the complex causal chain.

## Data Availability

The datasets presented in this article are not readily available because the data that support the findings of this study are available from the corresponding author upon reasonable request. Requests to access the datasets should be directed to chenzhuoheze@163.com.
